# Blood Lead Concentrations in Jamaican Children with and without Autism Spectrum Disorder

**DOI:** 10.3390/ijerph120100083

**Published:** 2014-12-23

**Authors:** Mohammad H. Rahbar, Maureen Samms-Vaughan, Aisha S. Dickerson, Katherine A. Loveland, Manouchehr Ardjomand-Hessabi, Jan Bressler, Sydonnie Shakespeare-Pellington, Megan L. Grove, Deborah A. Pearson, Eric Boerwinkle

**Affiliations:** 1 Division of Epidemiology, Human Genetics, and Environmental Sciences (EHGES), University of Texas School of Public Health at Houston, Houston, TX 77030, USA; E-Mail: Eric.Boerwinkle@uth.tmc.edu; 2 Division of Clinical and Translational Sciences, Department of Internal Medicine, University of Texas Medical School at Houston, Houston, TX 77030, USA; 3 Biostatistics/Epidemiology/Research Design (BERD) Component, Center for Clinical and Translational Sciences (CCTS), University of Texas Health Science Center at Houston, Houston, TX 77030, USA; E-Mails: Aisha.S.Dickerson@uth.tmc.edu (A.S.D.); Manouchehr.Hessabi@uth.tmc.edu (M.A.-H.); 4 Department of Child & Adolescent Health, The University of the West Indies (UWI), Mona Campus, Kingston 7, Jamaica; E-Mails: msammsvaughan@gmail.com (M.S.-V.); sydonniesp@gmail.com (S.S.-P.); 5 Department of Psychiatry and Behavioral Sciences, University of Texas Medical School at Houston, Houston, TX 77054, USA; E-Mails: Katherine.A.Loveland@uth.tmc.edu (K.A.L.); Deborah.A.Pearson@uth.tmc.edu (D.A.P.); 6 Human Genetics Center, University of Texas School of Public Health at Houston, Houston, TX 77030, USA; E-Mails: Jan.Bressler@uth.tmc.edu (J.B.); Megan.L.Grove@uth.tmc.edu (M.L.G.)

**Keywords:** autism spectrum disorder, blood lead concentrations, seafood, vegetables, fruits, Jamaica

## Abstract

Autism Spectrum Disorder (ASD) is a neurodevelopmental disorder manifesting by early childhood. Lead is a toxic metal shown to cause neurodevelopmental disorders in children. Several studies have investigated the possible association between exposure to lead and ASD, but their findings are conflicting. Using data from 100 ASD cases (2–8 years of age) and their age- and sex-matched typically developing controls, we investigated the association between blood lead concentrations (BLC) and ASD in Jamaican children. We administered a questionnaire to assess demographic and socioeconomic information as well as exposure to potential lead sources. We used General Linear Models (GLM) to assess the association of BLC with ASD status as well as with sources of exposure to lead. In univariable GLM, we found a significant difference between geometric mean blood lead concentrations of ASD cases and controls (2.25 μg/dL cases *vs.* 2.73 μg/dL controls, *p* < 0.05). However, after controlling for potential confounders, there were no significant differences between adjusted geometric mean blood lead concentrations of ASD cases and controls (2.55 μg/dL *vs.* 2.72 μg/dL, *p* = 0.64). Our results do not support an association between BLC and ASD in Jamaican children. We have identified significant confounders when assessing an association between ASD and BLC.

## 1. Introduction

Autism Spectrum Disorder (ASD) affects language development, communication, imagination, and social interactions. Repetitive, stereotyped behaviors are characteristic features of ASD [1,2] that manifest in early childhood [3,4,5]. The prevalence of ASD in the US population is about 1 in 68 [6] but in developing countries reliable estimates for the prevalence of ASD are rare. The etiology of ASD is believed to be multifactorial [7] and researchers believe that ASD is caused by the interplay of genes [8,9,10,11] and environmental factors [12,13,14,15].

Lead (Pb) belongs to the sulfhydryl-reactive metals that are the most toxic and insidious because they disrupt many biochemical and nutritional processes [16]. In fact, childhood lead poisoning is recognized as an important factor associated with neurodevelopmental impairment [17]. Lead is also shown to cause neurological, physiological, and behavioral disorders in children [17]. The US Centers for Disease Control and Prevention (CDC) defines blood lead concentration as “elevated” or “level of concern” if it is 10 μg/dL or greater [18]. However, it has been shown that even blood lead concentrations below 10 μg/dL are inversely associated with children’s intelligence quotient (IQ) scores at three and five years of age [19,20]. As a result of these findings, the CDC has recommended changes for the threshold of elevated blood lead concentrations to 5 μg/dL, but recommended not to use the term “level of concern” for this new threshold [21,22]. Regardless of cutoff values, no level of blood lead is considered safe, particularly due to its adverse effects on neurodevelopment in children [23,24,25].

Several studies have investigated the possible association between exposure to lead and ASD, but their findings are conflicting [26,27,28,29,30,31,32,33,34,35,36,37,38]. For example, a case-control study of 40 boys (4–8 years) with ASD and 40 non-affected age-matched typically developing (TD) boys from Kuwait reported increased levels of lead in the hair of children with ASD (median = 6.75 μg/g in cases *vs.* 3.20 μg/g in controls; *p* < 0.001) [27]. More recently, based on a case-control study in Saudi Arabia (25 children with ASD and 25 TD children), Blaurock-Busch *et al*. (2011) reported a significant difference between ASD cases and TD controls with respect to mean levels of lead in hair (*p* = 0.03) [32]. On the other hand, another study from Saudi Arabia that involved 52 children with ASD and 30 TD controls between 3 and 12 years of age reported that ASD cases had a significantly higher mean red blood cell lead concentration compared to TD controls (6.79 μg/dL *vs.* 4.73 μg/dL; *p* < 0.001) [38]. Lakshmi and Geetha (2010) reported a significant elevation in the levels of lead in hair samples from children with low functioning autism (LFA) (*p* < 0.001), medium functioning autism (MFA) (*p* < 0.001), and high functioning autism (HFA) (*p* < 0.01) compared to age- and sex-matched TD controls. This association was also seen in comparisons of lead levels in nails of children with LFA (*p* < 0.001) and controls in the same study [36]. Similarly, Adams *et al*. (2013) reported that the ASD cases had higher levels of lead in red blood cells (mean = 19 mcg/g in ASD cases *vs.* 13 mcg/g in controls; *p* = 0.002) [35]. In addition, Blaurock-Busch *et al*. (2012) reported a significant association between lead levels in hair and ASD severity scores in verbal communications (*p* = 0.02) and general impression (*p* < 0.008) in a study that involved 44 children with ASD from Saudi Arabia [33]. In contrast, a study from Dallas, Texas reported a significantly lower concentration of lead in the hair of 45 children with ASD (ages 1–5 years) compared with 45 gender-, age- and race-matched controls [28]. Moreover, another study suggested that children with ASD may have a poor heavy-metal-detoxifying mechanism, and as a result cannot excrete lead from their bodies compared to healthy controls (mean lead levels in urine = 1.19 μg/g creatinine in ASD cases *vs.* 4.63 μg/g creatinine in controls; *p* < 0.001) [29]. On the other hand, the Childhood Autism Risk from Genetics and Environment (CHARGE) study in California did not find any significant differences (*p* = 0.97) between mean blood lead concentrations of 2–5 year old children with ASD (n = 37) and those of TD controls (n = 15) [31]. Notable is the fact that none of the aforementioned studies adjusted their results to control for potential environmental exposures.

Previous studies have reported a high level of lead in the water [39] and in the soil of Jamaica [40]. Specifically, it has been reported that lead levels in Jamaican soil are about four times that of lead levels in some other parts of the world [39]. Previous studies also reported a significant correlation between levels of lead in the soil and root vegetables including carrots, onions, and radishes which were grown in areas with lead contaminated soil (r = 0.56; *p* < 0.001) [41]. A study conducted in Jamaica by Wright *et al*. (2012) investigated concentrations of nine residual metals in some Jamaican foods and reported that sweet potato had the highest concentration of lead (0.31 mg/kg) [42]. In addition, they reported a significant correlation (r ≥ 0.7) between soil lead levels and agricultural produce concentrations of lead [42]. Jamaica has very specific sources of environmental exposure to lead. In this study, we investigated whether there is an association between blood lead concentrations and ASD by comparing the geometric mean blood lead concentrations in children with and without ASD.

## 2. Materials and Methods

### 2.1. General Description

The Jamaican Autism Study is an age- and sex-matched case-control study that enrolled children age 2–8 years old from December 2009–March 2012, and investigated whether environmental exposures to several heavy metals, including lead, are associated with ASD. Information regarding the recruitment and assessment of ASD cases and TD controls has been reported previously [43,44,45,46,47,48]. In short, children listed in the University of the West Indies’ (UWI) Jamaica Autism Database, who were previously diagnosed as having ASD based on Diagnostic Statistical Manual of Mental Disorders (DSM-IV-TR) criteria [49] and the Childhood Autism Rating Scale (CARS) [50] were invited to participate for reassessment of their ASD status. The inclusion criteria for all children in the study were that each child must be born in Jamaica and be between 2 and 8 years of age at the time of enrollment. Parents of children listed in the UWI Jamaica Autism Database who met inclusion criteria were invited to participate in this study. Those who expressed interest and provided written informed consent for participation in the study were enrolled. For ascertainment of ASD status, we used standard algorithms developed for scoring the ADOS [51] and ADI-R and established thresholds [52]. Each ASD case was confirmed based on both the ADI-R and all three domains in the ADOS administered at the UWI Department of Child and Adolescent Health in Kingston, Jamaica by a trained senior psychologist to confirm diagnosis of ASD for the purposes of this research. For each case, an age- and sex-matched control (within six months of their matched cases) was identified from schools, well-child clinics, and community churches. The Lifetime Form of the Social Communication Questionnaire (SCQ) [53] was administered to the parents/guardians of TD control children to rule out symptoms of ASD using a cut-off point of 6, which is one standard deviation above the mean SCQ score of TD school children [54].

We also administered a pre-tested questionnaire to the parents/guardians of both ASD cases and TD controls to collect demographic and socioeconomic status (SES) information, parental education levels, and potential exposure to lead. Food frequency data represent current typical consumption of food items by children including types of vegetables, fruits, and seafood. Additionally, we collected information regarding children’s potential exposure to lead through pica, toys, and type of pots, pans, and dishes used by the family. Furthermore, we collected data regarding environmental exposure to lead through parental occupation, proximity of the child’s home to a high traffic road (whether or not the child’s home was within a quarter of a mile of a high traffic road), and whether the child lived in a home that is located within a mile of automobile battery repair shops, automobile battery recycling centers, or battery processing facilities [55,56]. At the end of the interviews, we collected 2 mL blood samples, which were frozen and stored at ‒20 °C until they were transported to the Michigan Department of Community Health (MDCH) Trace Metals Lab at ambient temperature on ice packs for trace metal analyses, including lead. Of the 150 case-control pairs enrolled in this study, only those children with both completed SES and food frequency questionnaires and measured blood lead concentrations were included in this analysis, totaling 100 pairs.

Institutional Review Boards (IRBs) of the University of Texas Health Science Center at Houston and UWI approved this study. The data presented herein represent analysis of 100 1:1 matched case-control pairs (200 children) for whom we had complete data.

### 2.2. Assessment of Lead Exposure

Lead exposure in humans can be measured through a variety of specimens including blood, bone, teeth, urine, hair, and nails. Lead in bone and tooth dentin and enamel represents cumulative or long-term exposure to lead [57]. Although lead can be excreted and measured in hair, washing and use of hair products can alter measured concentrations [58]. A study from Italy reported a significant correlation between blood lead concentrations and levels of lead in hair (r = 0.51) [59]. Additionally, urine excretion accounts for less than 1/6 of total excretion of absorbed lead, and is thus only indicative of very recent exposures [58]. Some studies of workers exposed to lead showed a positive correlation between levels of lead in blood and urine [60,61,62]. On the other hand, some studies reported that blood and urine lead concentrations are weakly related [63,64,65]. The correlation between lead in blood and urine depends on the duration of exposure [66]. Although blood lead concentration is a better predictor of more recent than long-term exposure, it is the most widely used biomarker for assessment of lead exposure. In this study, venous whole blood samples were assayed for lead by the Trace Metals Lab at MDCH, which is certified by the CDC for analysis of trace metals. MDCH followed a fully validated protocol for analyzing lead in blood samples with a detection limit of 0.3 μg/dL. However, none of the children in this study had blood lead concentrations below the limit of detection. All samples were diluted and analyzed on a PerkinElmer Elan DRC II inductively-coupled plasma mass spectrometer (PerkinElmer, Waltham, MA, USA).

### 2.3. Statistical Analysis

Descriptive analyses were conducted to compare demographic and socioeconomic status (SES) of ASD cases and TD controls. Since the distribution of blood lead concentrations is skewed, we transformed the data using the natural logarithm (ln) in order to produce a distribution that better approximated a normal distribution. The means of the ln transformed blood lead concentrations were transformed to their original scale (*i.e.*, μg/dL) by applying an exponential function, herein called geometric mean, that is, Exp. [Mean (lnPb)] = geometric mean. Similarly, geometric standard deviation (SD) of blood lead concentrations = Exp. {standard deviation of [Mean (lnPb)]}.

Since this is a 1:1 sex- and age-matched case-control study, we used General Linear Models (GLMs) with random effects to assess significant differences between the ASD cases and TD controls with respect to geometric mean blood lead concentrations. Moreover, we controlled for the effect of clustering caused due to matching by including 99 dummy variables (100 − 1 = 99, one pair serving as a referent category) to represent the 100 matched pairs as described in our previously published work [43,44,45,46,47,48]. Associations between the categorical exposure variables and ASD case status were assessed using Conditional Logistic Regression (CLR). Using GLMs, we also assessed the association between log transformed blood lead concentrations and various exposure variables, including pica (e.g., habitually ate mud) and frequency of eating various kinds of seafood or fish (e.g., shellfish), root vegetables (e.g., sweet potato), and fruits (e.g., ackee). In addition, we assessed the association between blood lead concentrations and types of pots, pans and dishes (e.g., Teflon) used for cooking and eating, child’s exposure to lead containing toys, maternal and paternal age at child’s birth, parental education levels, parish of child’s birth, and SES through car ownership. Since maternal and paternal levels of education are significantly correlated, we created a binary variable indicating whether both parents had education up to high school or at least one of the parents obtained education beyond high school in order to minimize any potential effects of multicollinearity. Moreover, since Lalor *et al*. (2007) [67] reported that the Kingston corporate area had higher blood lead concentrations, we created a binary variable indicating whether the child was born in the Kingston parish. These variables were used in the CLR and GLM analyses. Before fitting a multivariable GLM to investigate the relationship between blood lead concentrations and ASD status, we identified potential confounders that were included in our final multivariable model. Selection of potential confounders was based on *a priori* measures (e.g., SES based on car ownership by the family) as well as potential sources of lead exposures, including consumption of root vegetables, leafy vegetables, fruits, and seafood, and variables found to be associated with both ASD case status and blood lead concentrations. For this we identified variables that were potentially associated with ASD case status based on *p* < 0.25 in the CLR model and potentially associated with blood lead concentrations if *p* < 0.25 in the GLM. The covariates were considered to be potential confounders if they changed the regression coefficient by >10% [68]. Since in the context of assessing associations between blood lead concentrations and ASD status dietary factors could be considered as confounders [69], we fit two multivariable GLMs. The first multivariable GLM only controlled for SES as well as sociodemographic indicators. The second multivariable GLM not only controlled for SES and sociodemographic indicators, but also controlled for various dietary factors and other potentially confounding variables [70,71]. After adjusting for these potential confounders in both multivariable GLMs, we calculated the adjusted geometric mean blood lead concentrations for children with and without ASD. We also tested for potential interactions between ASD status and other covariates in the final multivariable GLM. Moreover, we conducted additional stratified analyses to investigate associations between exposure variables and geometric mean lead levels by ASD status using *t*-tests. All statistical analyses were conducted at 5% level of significance using SAS 9.3 [72].

## 3. Results

Children in the ASD group had a mean age of 68.4 months, while the mean age for children in the control group was 69.3 months. Nearly all of the ASD cases (93.0%) and TD controls (99.0%) were Afro-Caribbean and 85.0% of the ASD cases and TD controls were male. Less than half of children (44.5%) in our study were born in the Kingston parish, but the majority of TD controls (70.0%) were born in the Kingston parish compared to 19.0% of ASD cases. Paternal education level was significantly higher in the ASD case group compared to TD controls (45.5% of fathers in the case group had education beyond high school compared to 12.4 % in the control group; *p* < 0.01). Similarly, a significantly higher level of education was attained by mothers in the ASD case group compared to the TD controls (47.0% of mothers in the ASD case group had education beyond high school *vs.* 23.5% in the control group, *p* < 0.01). A comparison of the assets owned by the ASD case families revealed that the SES of the case families was significantly higher than that of the TD control group. Demographic and SES characteristics of the ASD cases and matched TD controls are reported in Table 1.

**Table 1:** Demographic and socioeconomic characteristics of children and their parents by ASD case status (200 children or 100 matched pairs).

Variables	Categories	Case (n = 100) N (%)	Control (n = 100) N (%)	*p*-Value
**Sex of child**	Male	85 (85.0)	85 (85.0)	1.00
**Age of child** (months)	Age < 48	16 (16.0)	13 (13.0)	0.64
	48 ≤ age < 72	46 (46.0)	47 (47.0)	
	Age ≥ 72	38 (38.0)	40 (40.0)	
**Place of child’s birth**	Kingston parish	19 (19.0)	70 (70.0)	<0.01
	Other areas	81 (81.0)	30 (30.0)	
**Maternal age** *^a^* (at child’s birth)	<35 years	76 (76.0)	84 (88.4)	0.02
	≥35 years	24 (24.0)	11 (11.6)	
**Maternal education** *^b^* (at child’s birth)	Up to high school ^✝^	53 (53.0)	75 (76.5)	<0.01
	Beyond high school ^✝✝^	47 (47.0)	23 (23.5)	
**Paternal age** *^c^* (at child’s birth)	<35 years	47 (48.5)	66 (77.1)	<0.01
	≥35 years	50 (51.5)	26 (28.3)	
**Paternal education** ^*d*^ (at child’s birth)	Up to high school ^✝^	53 (54.6)	85 (87.6)	<0.01
	Beyond high school ^✝✝^	44 (45.4)	12 (12.4)	
**Number of children in the household** (Age ≤ 18 years)	1–2	80 (80.0)	53 (53.0)	<0.01
	≥3	20 (20.0)	47 (47.0)	
**Number of adultsin the household** (Age > 18 years) *^e^*	1–2	64 (64.0)	60 (60.6)	0.66
	≥3	36 (36.0)	39 (39.4)	
**Assets owned by the family**	TV	99 (99.0)	94 (94.0)	0.10
	Refrigerator	97 (97.0)	85 (85.0)	<0.01
	Freezer	12 (12.0)	19 (19.0)	0.17
	Living room set	85 (85.0)	47 (47.0)	<0.01
	Washing machine	76 (76.0)	53 (53.0)	<0.01
	Cars or other vehicle	68 (68.0)	37 (37.0)	<0.01
	Telephone/Cell phone	99 (99.0)	99 (99.0)	1.00
	Cable/Satellite connection	65 (65.0)	36 (36.0)	<0.01

Notes: ^✝^ Up to high school education means attended Primary/Jr. Secondary, and Secondary/High/Technical schools; ^✝✝^ Beyond high school education means attended a Vocational, Tertiary College, or University; *^a^* Maternal age was missing for 5 controls; *^b^* Maternal education was missing for 2 controls; *^c^* Paternal age was missing for 3 cases and 8 controls; *^d^* Paternal education was missing for 3 cases and 3 controls; *^e^* Number of adults in the household was missing for 1 control family.

In the process of identifying potential confounding variables, the CLR analyses also revealed that parental education levels were significantly higher in the ASD case group compared with the TD control group, (Matched Odds Ratio (MOR) = 3.50, 95% CI (1.84, 6.65); *p* < 0.01). In addition, compared to the TD control group, there was a higher proportion of children in the ASD case group who exhibited pica (habitually ate mud) (MOR = 3.00, 95% CI (1.28, 7.06); *p* = 0.01); lived near a high traffic road (MOR = 3.73, 95% CI (1.92, 7.26); *p* < 0.01); and played with battery-operated toys (MOR = 4.38, 95% CI (2.03, 9.43); *p* < 0.01) and electronic toys (MOR = 4.86, 95% CI (2.28, 10.43); *p* < 0.01). A similar comparison revealed that a significantly lower proportion of ASD cases reported eating sardine or mackerel (MOR = 0.26, 95% CI (0.11, 0.64); *p* < 0.01) and cabbage (MOR = 0.15, 95% CI (0.06, 0.38); *p* < 0.01). Comparisons of other dietary exposures between children with and without ASD are displayed in Table 2.

**Table 2:** Association between potential confounders and ASD case status using Conditional Logistic Regression (CLR) (200 children or 100 matched pairs).

Exposure Variables	Category		ASD Case N (%)	TD Control N (%)	Matched OR (MOR)	95% CI for MOR	*p*-Value
**Parental education levels** *^a^* (at child’s birth)	At least one of the parents had education beyond high school		64 (66.0)	30 (31.6)	3.50	(1.84, 6.65)	<0.01
**Socioeconomic status** (SES)	Higher SES (own a car)		68 (68.0)	37 (37.0)	3.58	(1.90, 6.80)	<0.01
**Source of drinking water** *^b^*	Piped water		94 (94.0)	95 (96.0)	0.67	(0.19, 2.36)	0.53
**Source of water for cooking** *^c^*	Piped water		94 (94.0)	95 (96.0)	0.67	(0.19, 2.36)	0.53
**Pica** (habitually put items in the mouth)	Mud		21 (21.0)	7 (7.0)	3.00	(1.28, 7.06)	0.01
	Paint chips **		5 (5.0)	1 (1.0)	NR	NR	NR
**Living near a high traffic road** *^d^*			59 (59.6)	29 (29.0)	3.73	(1.92, 7.26)	<0.01
**Home environment**	Living with adults whose job involve battery repair shop or battery recycling or processing *^e^*		7 (7.1)	2 (2.0)	3.50	(0.73, 16.85)	0.19
	Living in a house with paint peeling or chipping off*^ f^*		29 (29.3)	25 (25.3)	1.19	(0.67, 2.13)	0.56
	Living with adults whose job involve construction		6 (6.0)	11 (11.0)	0.50	(0.17, 1.46)	0.21
**Types of toys the child plays with**	Plastic *^g^*		97 (97.0)	90 (90.9)	3.00	(0.81, 11.08)	0.10
	Electronic *^h^*		71 (71.0)	40 (40.4)	4.86	(2.28, 10.43)	<0.01
	Battery operated		76 (79.0)	52 (52.0)	4.38	(2.03, 9.43)	<0.01
	Stuffed		76 (76.0)	66 (66.0)	1.71	(0.89, 3.31)	0.11
**Types of pots, pans, and dishes used at home**	Cast iron *^i^*		53 (53.0)	42 (42.4)	1.56	(0.86, 2.81)	0.14
	Steel coated with porcelain enamel*^ j^*		17 (17.0)	5 (5.1)	4.00	(1.34, 11.96)	0.01
	Teflon *^k^*		48 (48.5)	24 (24.0)	3.01	(1.61, 5.91)	<0.01
	Ceramic		80 (80.0)	72 (72.0)	1.89	(0.84, 4.24)	0.12
	Aluminum		94 (94.0)	84 (84.0)	3.50	(1.15, 10.63)	0.03
**Fruits and vegetables consumption** *^l^*	Root vegetables	A. Yam, sweet potato, or dasheen	73 (73.0)	82 (82.8)	0.52	(0.25, 1.07)	0.08
		B. Carrot or pumpkin	86 (86.0)	98 (99.0)	0.08	(0.01, 0.59)	0.01
	Leafy vegetables	A. Lettuce	47 (47.0)	62 (62.6)	0.57	(0.33, 0.97)	0.04
		B. Callaloo, broccoli, or pakchoi	72 (72.0)	94 (94.9)	0.18	(0.07, 0.46)	<0.01
		C. Cabbage	66 (66.0)	94 (94.9)	0.15	(0.06, 0.38)	<0.01
	Fruits	Tomatoes	62 (62.0)	85 (85.9)	0.23	(0.10, 0.51)	<0.01
		Ackee	58 (58.0)	92 (92.9)	0.06	(0.01, 0.23)	<0.01
		Avocado	29 (29.0)	68 (68.7)	0.19	(0.09, 0.38)	<0.01
		Green banana	67 (67.0)	90 (90.9)	0.27	(0.13, 0.57)	<0.01
		Fried plantains	70 (70.0)	89 (89.9)	0.17	(0.06, 0.48)	<0.01
**Seafood consumption**	Ate salt water fish		77 (77.0)	89 (89.0)	0.40	(0.18, 0.91)	0.03
	Ate fresh water fish (Pond fish, Tilapia)		46 (46.0)	52 (52.0)	0.75	(0.41, 1.38)	0.36
	Ate sardine, mackerel (Canned fish)		75 (75.0)	92 (92.0)	0.26	(0.11, 0.64)	<0.01
	Ate tuna (Canned fish)		31 (31.0)	44 (44.0)	0.55	(0.30, 1.02)	0.06
	Ate salt fish (Pickled mackerel)		70 (70.0)	93 (93.0)	0.15	(0.05, 0.42)	<0.01
	Ate shellfish (Lobsters, Crabs)		7 (7.0)	14 (14.0)	0.42	(0.15, 1.18)	0.10
	Ate shrimp		19 (19.0)	27 (27.0)	0.62	(0.31, 1.24)	0.17

Notes: NR = Not reported due to unstable estimates caused by a limited number of observation in at least one of the cells; *^a^* Parental education levels were missing for 3 cases and 5 controls; *^b^* Source of drinking water was missing for 1 control; *^c^* Source of water for cooking was missing for 1 control; *^d^* Living near a high traffic road was missing for 1 case; *^e^* Living with adult whose job involves with battery repair shop or battery recycling or processing was missing for 1 case; *^f^* Living in a house with paint peeling or chipping off was missing for 1 case and 1 control; *^g^* Plastic toys was missing for 1control; *^h^* Electronic toys was missing for 1control; *^i^* Cast iron was missing for 1control; *^j^* Steel coated with porcelain enamel was missing for 1control; *^k^* Teflon was missing for 1 case; *^l^* For all variables under fruits and vegetables consumption data were missing for 1 control.

A comparison of geometric mean blood lead concentrations between children who had different levels and types of exposures revealed the following findings. In the univariable analysis, compared with children who did not have pica (in our situation it only refers to the habit of eating mud), children who exhibited pica had a significantly higher geometric mean blood lead concentration (3.84 μg/dL *vs.* 2.31 μg/dL; *p* < 0.01). Children who ate shellfish had a higher geometric mean blood lead concentration compared with those who did not (3.76 μg/dL *vs.* 2.36 μg/dL; *p* = 0.05). Though marginally significant, we found a difference between geometric mean blood lead concentrations of children whose family consumed fresh water fish and those who did not (2.85 μg/dL *vs.* 2.17 μg/dL, respectively; *p* = 0.07). Comparisons of geometric mean blood lead concentrations for other exposure variables are displayed in Table 3.

**Table 3:** Factors associated with blood lead concentrations based on univariable General Linear Models (100 matched pairs or 200 children).

Exposure Variables	Category		Univariable Analysis				
			Yes		No		*p-*Value
			Mean ± SD (μg/dL)	N	Mean ± SD (μg/dL)	N	
**Place of child’s birth**	Kingston parish		2.82 ± 1.99	89	2.24 ± 2.00	111	0.15
**Paternal age** (at child’s birth)	≥35 years		2.16 ± 1.98	76	2.68 ± 2.02	113	0.17
**Maternal age** (at child’s birth)	≥35 years		2.10 ± 2.04	35	2.59 ± 2.06	160	0.26
**Parental education levels** (at child’s birth)	At least one of the parents had education beyond high school		2.43 ± 2.04	94	2.54 ± 2.02	98	0.78
**Socioeconomic status (SES)**	High SES (own a car)		2.46 ± 2.10	105	2.50 ± 1.97	95	0.90
**Source of drinking water**	Piped water		2.51 ± 2.07	189	1.96 ± 1.60	10	0.42
**Source of cooking water**	Piped water		2.51 ± 2.07	189	1.96 ± 1.60	10	0.42
**Pica** (habitually put items in the mouth)	Habitually ate mud		3.84 ± 2.04	28	2.31 ± 1.98	172	<0.01
**Living near a high traffic road**			2.34 ± 2.24	88	2.59 ± 2.36	111	0.51
**Home environment**	Living with adults whose job involve battery repair shop or battery recycling or processing		2.46 ± 2.03	9	2.48 ± 2.33	190	0.98
	Living in a house with paint peeling or chipping off		2.30 ± 2.02	54	2.58 ± 2.11	144	0.43
	Living with adults whose job involve construction		1.74 ± 2.07	17	2.56 ± 1.93	183	0.13
**Types of toys the child plays with**	Plastic		2.47 ± 1.90	187	2.88 ± 2.06	12	0.59
	Electronic		2.22 ± 1.99	111	2.79 ± 2.02	88	0.16
	Battery operated		2.33 ± 2.03	131	2.80 ± 2.03	69	0.26
	Stuffed		2.33 ± 2.01	142	2.89 ± 2.15	58	0.18
**Types of pots, pans, and dishes used at home**	Cast iron		2.47 ± 2.00	95	2.54 ± 2.12	104	0.84
	Steel coated with porcelain enamel		2.37 ± 2.05	22	2.50 ± 2.09	177	0.82
	Teflon		2.03 ± 2.01	72	2.76 ± 2.08	127	0.04
	Ceramic		2.60 ± 2.02	152	2.12 ± 1.98	48	0.29
	Aluminum		2.45 ± 1.92	178	2.73 ± 2.07	22	0.65
**Fruits and vegetables consumption**	Root vegetables	A. Yam, sweet potato, or dasheen	2.51 ± 2.30	155	2.48 ± 2.05	44	0.95
		B. Carrot or pumpkin	2.48 ± 2.04	184	2.80 ± 2.09	15	0.66
	Leafy vegetables	A. Lettuce	2.55 ± 2.09	109	2.44 ± 2.03	90	0.72
		B. Callaloo, broccoli, or pakchoi	2.50 ± 1.98	166	2.51 ± 2.30	33	0.98
		C. Cabbage	2.48 ± 1.98	160	2.58 ± 2.45	39	0.82
	Fruits	Tomatoes	2.56 ± 1.99	147	2.33 ± 2.22	52	0.59
		Ackee	2.63 ± 1.99	150	2.15 ± 2.23	49	0.29
		Avocado	2.48 ± 1.90	97	2.52 ± 2.21	102	0.90
		Green banana	2.55 ± 2.01	157	2.32 ± 2.20	42	0.55
		Fried plantains	2.62 ± 2.46	159	2.08 ± 1.96	42	0.25
**Seafood consumption**	Ate salt water fish		2.56 ± 2.19	166	2.12 ± 2.03	34	0.31
	Ate fresh water fish (Pond fish, Tilapia)		2.85 ± 2.07	98	2.17 ± 2.03	102	0.07
	Ate sardine, mackerel (Canned fish)		2.47 ± 2.03	167	2.50 ± 2.19	33	0.95
	Ate tuna (Canned fish)		2.30 ± 1.78	75	2.59 ± 2.21	125	0.43
	Ate salt fish (Pickled mackerel)		2.54 ± 2.42	163	2.21 ± 1.97	37	0.47
	Ate shellfish (Lobsters, Crabs)		3.76 ± 1.88	21	2.36 ± 2.07	179	0.05
	Ate shrimp		1.98 ± 1.93	46	2.65 ± 2.09	154	0.08

Notes: Mean indicates the geometric mean = Exp. [Mean (lnPb)]; Standard deviation (SD) indicates the geometric standard deviation= Exp. {standard deviation of [Mean (lnPb)]}; The **Yes** column includes subjects who met the category specified in front of each exposure variable; The **No** column includes subjects who did not meet the category specified in front of each exposure variable; Information regarding the missing values for all variables in this table has already been reported in Table 1 and Table 2.

Our results from the unadjusted GLM showed a higher geometric mean blood lead concentration for TD controls in comparison to ASD cases (2.73 µg/dL *vs.* 2.25 µg/dL; *p* < 0.05). In separate GLMs after controlling for potential confounding sociodemographic indicators including SES (*i.e.*, car ownership by the family), parental education levels, place of child’s birth, and maternal age at the child’s birth, we did not find a significant association (2.26 µg/dL *vs.* 2.43 µg/dL; *p* = 0.62) between blood lead concentrations and ASD status. Additionally, as shown in Table 4, after controlling for dietary exposures, including consumption of shellfish (lobsters, crabs), and use of Teflon (pots, pans, and dishes) for cooking, there was also no statistically significant association between blood lead concentrations and ASD status (2.72 µg/dL *vs.* 2.55 µg/dL; *p* = 0.64). Details regarding the association of various exposure variables and blood lead concentrations are shown in Table 4.

**Table 4:** Unadjusted and adjusted mean blood lead concentrations for 100 ASD cases and their 1:1 matched controls based on the GLMs.

Models	Mean ± SD (μg/dL)		*p-*Value
	ASD Cases	TD Controls	
Unadjusted	2.25 ± 2.23	2.73 ± 1.85	<0.05
Adjusted *^a^*	2.26 ± 2.03	2.43 ± 2.03	0.62
Adjusted *^b^*	2.55 ± 2.02	2.72 ± 2.02	0.64

Notes: Mean indicates the geometric mean = Exp. [Mean (lnPb)]; Standard deviation (SD) indicates the geometric standard deviation= Exp. {standard deviation of [Mean (lnPb)]}; For adjusted models Standard deviation (SD) indicates the geometric standard deviation which is assumed to be the same for ASD cases and TD controls= Exp. [$\sqrt{\text{Mean squared error}}$]; *^a^* Factors adjusted for include: maternal age, parental education levels, parish at child’s birth, SES (*i.e.*, car ownership by the family); *^b^* Factors adjusted for include: maternal age, parental education levels, parish at child’s birth, SES (*i.e.*, car ownership by the family), consumption of shellfish (lobsters, crabs), and Teflon (pots, pans, and dishes) for cooking.

Stratified analysis of associations between potential exposure variables and geometric mean blood lead levels showed that in ASD cases, children born in Kingston parish had higher mean blood lead concentrations than ASD cases who were born in other parishes (3.29 µg/dL *vs.* 2.05 µg/dL; *p* = 0.02). A similar association was observed for TD control children who were born in Kingston parish having a significantly higher mean blood lead concentration than the TD control children born in other parishes (3.04 µg/dL *vs.* 2.14 µg/dL; *p* < 0.01). In contrast, analysis of associations between maternal age at birth and mean blood lead concentrations revealed that in ASD cases, the mean blood lead concentration was significantly lower in ASD cases whose mother was over 35 years of age at birth of the child as compared to ASD cases whose mother was at most 35 years of age at birth of the child (1.70 µg/dL *vs.* 2.46 µg/dL; *p* < 0.05). On the other hand, mean blood lead concentrations of TD controls were not significantly different between the two aforementioned categories of maternal age at birth of the child (*p* = 0.75). Also, we tested for possible interactions between individual covariates in the final multivariable model and ASD status, and found no significant interactions (results not shown). More details regarding these stratified analyses are shown in Table 5.

**Table 5:** Associations between individual covariates in the final multivariable GLM and geometric mean lead levels stratified by ASD status using *t*-tests (200 children or 100 pairs).

Exposure Variables	Category	*t*-Test									
		n = 100 ASD Cases					n = 100 TD Controls				
		Yes		No		*p*-Value	Yes		No		*p*-Value
		N	Mean ± SD (μg/dL)	N	Mean± SD (μg/dL)		N	Mean ± SD (μg/dL)	N	Mean ± SD (μg/dL)	
**Place of child’s birth**	Kingston parish	19	3.29 ± 2.59	81	2.06 ± 2.09	0.02	70	3.04 ± 1.83	30	2.14 ± 1.79	<0.01
**Maternal age** (at child’s birth)	>35 years	24	1.70 ± 2.17	76	2.46 ± 2.21	<0.05	11	2.88 ± 1.54	84	2.70 ± 1.88	0.75
**Parental education levels** (at child’s birth)	At least one of the parents had education beyond high school	64	2.08 ± 2.14	33	2.56 ± 2.31	0.22	30	2.35 ± 1.83	65	2.92 ± 1.88	0.12
**Socioeconomic status (SES)**	High SES (family owns a car)	68	2.11 ± 2.32	32	2.57 ± 2.01	0.25	37	2.44 ± 1.65	63	2.92 ± 1.96	0.16
**Types of pots, pans, and dishes used at home**	Teflon	48	1.97 ± 2.19	51	2.53 ± 2.26	0.12	24	2.29 ± 1.88	76	2.89 ± 1.83	0.10
**Seafood Consumption**	Ate shellfish (Lobsters, crabs)	7	1.81 ± 1.56	93	2.28 ± 2.27	0.46	14	3.58 ± 1.81	86	2.62 ± 1.85	0.08

Notes: Mean Pb indicates the geometric mean = Exp. [Mean (lnPb)]; Standard deviation (SD) indicates the geometric standard deviation= Exp. {standard deviation of [Mean (lnPb)]}; The **Yes** column includes subjects who met the category specified in front of each exposure variable; The **No** column includes subjects who did not meet the category specified in front of each exposure variable; Information regarding the missing values for all variables in this table has already been reported in Table 1, Table 2 and Table 3; *p*-value is based on *t*-tests that compares mean blood lead concentrations between the Yes and the No categories of the exposure variables.

## 4. Discussion

### 4.1. Blood Lead Concentrations and ASD

Our results do not support an association between postnatal blood lead concentration measured in Jamaican children 2–8 years of age and ASD case status. Although, in the univariable analysis we found a significant association between blood lead concentrations and ASD status, with lower blood lead concentrations in ASD cases (2.25 µg/dL for ASD *vs.* 2.73 µg/dL for TD; *p* < 0.05), when we adjusted for potential confounding sociodemographic indicators and environmental exposures, there was not sufficient evidence to indicate an association between blood lead concentrations and ASD status in children. Specifically, when we adjusted for maternal age, parental education levels, parish at child’s birth, and SES (*i.e.*, car ownership by the family) we did not find a significant difference in the adjusted geometric mean blood concentrations of children with and without ASD (2.26 µg/dL for ASD *vs.* 2.43 µg/dL for TD controls; *p* = 0.62). Furthermore, when we adjusted for all potential confounders that included maternal age, parental education levels, parish at child’s birth, SES (*i.e.*, car ownership by the family), consumption of shellfish (lobsters, crabs), and Teflon (pots, pans, and dishes) used for cooking, we still did not find a significant difference in the adjusted geometric mean blood concentrations of children with and without ASD (2.55 µg/dL for ASD *vs.* 2.72 µg/dL for TD controls; *p* = 0.64).

Our univariable results are consistent with findings from univariable findings of several other studies that reported lower levels of lead in children with ASD compared with TD controls. For example, Kern *et al*. (2007) reported significantly lower (*p* < 0.05) lead levels in hair of children with ASD compared to a group of 45 age-, sex-, and ethnicity-matched TD controls from Dallas (TX, USA) [28]. Also, Yorbik *et al*. (2010) reported a significantly (*p* < 0.001) lower mean concentration of lead in urine of 30 children with ASD compared to a group of 20 TD children, of age 3–12 years [29]. However, we acknowledge that because the previously mentioned studies used different biomarkers for assessment of lead, our results may not be entirely comparable.

On the other hand, our univariable results are in contrast with findings from those of several other studies that reported no associations between lead levels and ASD or found higher levels of lead in children with ASD compared with TD controls. For example, the CHARGE study from California reported no significant association between mean blood lead concentrations and ASD status (1.30 µg/dL for TD *vs.* 1.30 µg/dL; *p* = 0.97) [31]. An age- and sex-matched case-control study in Saudi Arabia reported significantly higher levels of lead in hair (*p* = 0.03) and urine (*p* = 0.004) [32]. Similarly, Fido and Al-Saad (2005) reported significantly higher levels of lead in hair of children with ASD than in TD controls in Kuwait (median = 6.75 μg/g in ASD cases *vs.* 3.20 μg/g in TD controls; *p* < 0.001) [27]. In addition, El-Ansary *et al.* (2011) reported higher mean lead concentrations in plasma of ASD children compared to a group of healthy age-matched controls in Saudi Arabia [37]. Another study by Al-Farsi *et al*. (2013) also reported higher levels of lead in hair of ASD cases compared with a group of matched control children from Oman (median = 12.2 µg/g for ASD *vs.* 6.2 µg/g for TD controls; *p* < 0.05) [73]. More recently, a study by Alabdali *et al.* (2014) reported a significantly higher mean red blood cell lead concentration in ASD cases compared to TD controls (6.79 μg/dL *vs.* 4.73 μg/dL; *p* < 0.001) [38]. Although, some of the aforementioned studies collected information regarding SES and dietary intake of children [73], none of these studies that reported an association between lead and ASD status have controlled for potential confounding effects due to differences in the SES or diets of children with and without an ASD.

### 4.2. Role of SES and Sociodemographic Indicators as Potential Confounders

Socioeconomic status and sociodemographic indicators, such as parental education, occupation, or income are strongly correlated with child development. In developmental disabilities, SES is often reported to have an inverse association [74,75]. However, for ASD, SES has been shown to be higher in children with an ASD diagnosis [76,77]. For example, several studies have reported that ASD is diagnosed more frequently in children of parents with more education [44,76,78,79,80,81,82,83]. Additionally, previous studies have reported a lower rate of ASD diagnosis and prevalence in groups with lower income [77,82,84]. On the other hand, Larsson *et al*. (2005) showed no statistically significant association between risk of ASD and socioeconomic status [85]. In addition, children with lower SES have been shown to have more exposure to chemical contaminants [86] including reported associations with increased blood lead concentrations in children with lower SES [87,88]. In our study, although we observed a significant association between ASD status and SES as well as sociodemographic indicators (maternal age, parental education levels, car ownership by the family, and parish at child’s birth), we did not find a significant association between the blood lead concentrations and the aforementioned SES and sociodemographic indicators. Nevertheless, we selected these SES as well as sociodemographic indicators as potential confounders based on *a priori* studies. No other studies, to our knowledge, investigating blood lead concentrations and ASD examined SES indicators as potential confounders [28,29,31,32,73].

### 4.3. Role of Dietary Factors as Potential Confounders

Some studies showed that dietary factors such as vegetables can be a source of lead exposure [41]. Furthermore, seafood consumption has also been implicated as a major source of exposure to lead [58,89,90,91,92]. We found that children who consumed shellfish (lobster, crabs) had higher geometric mean blood lead concentrations than those who did not eat shellfish (*p* = 0.05); however, we did not observe this association for vegetables and fruits. Additionally, we attempted to control for potential exposure to lead through cooking materials by adjusting for the types of pots, pans, and dishes used for cooking, such as Teflon; however, we found an inverse association between usage of Teflon and blood lead concentrations in children. This may be due to only ascertaining if families used Teflon for cooking, but not asking the frequency of use, which may have yielded unexpected results.

Due to unstable estimates caused by limited data in some categories, pica (habitually eating mud) was not included in multivariable analyses. A comparison of the results in our univariable and multivariable models suggests a significant confounding effect by diet and SES as well as sociodemographic indicators. For example, it is well established that children with ASD have a higher incidence of gastrointestinal (GI) problems (70% *vs.* 28%) [93], a higher incidence of constipation (33.9% *vs.* 17.6%) [94], and feeding issues which may result in food selectivity for children with ASD (24.5% *vs.* 16.1%) compared to TD children [95]. Therefore, we presented results from two multivariable models. One multivariable model adjusted for several potentially confounding SES and sociodemographic indicators while the second multivariable model adjusted for the same SES and sociodemographic indicators along with dietary exposure factors, including consumption of seafood (lobster, crab), and use of Teflon (pots, pans, and dishes) for cooking. The differences seen between our results and those of previously mentioned studies may be attributed to their not adjusting for potential confounding factors.

### 4.4. Stratified Analyses, Estimated Effect Size, Sample Size, and Statistical Power

Since our main findings indicate a lack of an additive effect of blood lead concentrations in ASD, we conducted additional analyses to rule out the possibility of interactions between ASD status and other covariates used in the final multivariable GLM. First, assessment of the aforementioned interaction in the final multivariable GLM did not result in any significant interactions (all *p*-values for interaction ≥0.49). We also compared the mean blood concentrations in various categories of exposure variables, stratified by ASD case status. These results did not suggest any potential for obtaining misleading parameter estimates for the regression parameters in terms of availability of sufficient data in various categories of exposure variables, separately by ASD cases and the TD controls. Additionally, based on the available 100 pairs of children, we have at least 80% power to detect small effect sizes (effect size greater than or equal to 0.28*SD of blood lead concentrations) between ASD cases and TD controls at 5% level of significance. For examples, since the standard deviation of blood lead concentrations is 2.02 µg/dL in the TD control group, our sample of 100 pairs will be able to detect a mean difference of 0.57 µg/dL (*i.e.*, 0.28*2.02 = 0.57) with 80% probability at 5% level of significance if such difference exists between the two populations of ASD cases and TD controls. Collectively, these findings suggest lack of a significant association between postnatal blood lead concentrations and ASD.

## 5. Limitations

We acknowledge several limitations in this study. First, since recruitment of the TD control children was more difficult than the ASD cases, a higher proportion of the TD control children were selected from the Kingston parish. Additionally, several limitations in this study have been reported previously [45]. For example, our TD controls belonged to a lower SES group than our ASD cases due to difficulty in recruitment because of the requirement of blood draw from the TD controls, particularly if the parents/guardians of the TD children had to miss their school in order to participate in this study. Even though some TD children were given an opportunity to be evaluated during weekends, there were SES differences between the ASD cases and the TD control groups. Previous studies have also suggested that ASD reporting is often higher in SES groups [44,76,77,79,80,81,82,83,84,96]. Thus, it is also possible that the lower SES seen in our control group is more representative of the Jamaican demographic, while our cases are from a higher SES group in Jamaica. However, since we have adjusted our results by SES and sociodemographic variables, we believe potential confounding effects of these variables has been accounted for in our multivariable GLMs. We also acknowledge that the blood lead concentrations in this study represent lead exposure only during the postnatal period, and we did not collect information regarding lead exposures during the perinatal and prenatal periods. Furthermore, the postnatal exposure to lead through diet may not necessarily represent lead exposure through diet during a time that may be causally related to ASD. We also could not assess potential changes in exposure conditions due to relocation of the mother during pregnancy, or of the child between birth and the time of assessment.

## 6. Conclusions

In this study, we found no significant association between ASD and postnatal blood lead concentrations in Jamaican children. We have identified significant confounders when assessing a possible association between ASD and blood lead concentrations in Jamaican children. After adjusting for sociodemographic factors, such as maternal age at child’s birth, parental education levels, parish at child’s birth, and SES (*i.e.*, car ownership by the family) in one multivariable model, and adjusting for the same sociodemographic factors along with dietary and other exposures to lead, including consumption of seafood (lobster, crab), and use of Teflon (pots, pans, and dishes) for cooking in a separate multivariable GLM, there was no significant association between ASD and blood lead concentrations in Jamaican children. Additionally, we have shown that SES and dietary habits may differ between ASD cases and TD controls. Although previous studies have shown associations between lead levels and ASD status as well as differences in lead metabolism between ASD cases and TD controls, the results from this study indicate that these reported relationships may have been impacted by sociodemographic indicators and dietary factors. Thus, future studies should further investigate how SES and nutrition may alter observed lead levels, and how these differences in SES and diet between ASD cases and TD controls can confound results for tests of association between lead levels and ASD case status.

## References

[1] Rapin I. (1997). Autism. N. Engl. J. Med..

[2] Newschaffer C.J., Croen L.A., Daniels J., Giarelli E., Grether J.K., Levy S.E., Mandell D.S., Miller L.A., Pinto-Martin J., Reaven J. (2007). The epidemiology of autism spectrum disorders. Annu. Rev. Public Health.

[3] Volkmar F., Chawarska K., Klin A. (2005). Autism in infancy and early childhood. Annu. Rev. Psychol..

[4] Volkmar F.R., Chawarska K. (2008). Autism in infants: An update. World Psychiatry.

[5] Genuis S.J. (2009). Is autism reversible?. Acta Paediatr..

[6] Autism and Developmental Disabilities Monitoring Network Surveillance Year 2010 Principal Investigators, Centers for Disease Control and Prevention (CDC) (2014). Prevalence of autism spectrum disorder among children aged 8 years—Autism and developmental disabilities monitoring network, 11 sites, United States, 2010. MMWR Surveill Summ..

[7] Gardener H., Spiegelman D., Buka S.L. (2011). Perinatal and neonatal risk factors for autism:A comprehensive meta-analysis. Pediatrics.

[8] Kumar R., Christian S. (2009). Genetics of autism spectrum disorders. Curr. Neurol. Neurosci. Rep..

[9] Bowers K., Li Q., Bressler J., Avramopoulos D., Newschaffer C., Fallin M.D. (2011). Glutathione pathway gene variation and risk of autism spectrum disorders. J. Neurodev. Disord..

[10] Buxbaum J.D., Hof P.R. (2011). The emerging neuroscience of autism spectrum disorders. Brain Res..

[11] Bill B.R., Geschwind D.H. (2009). Genetic advances in autism: Heterogeneity and convergence on shared pathways. Curr. Opin. Genet. Dev..

[12] Herbert M.R. (2010). Contributions of the environment and environmentally vulnerable physiology to autism spectrum disorders. Curr. Opin. Neurol..

[13] Landrigan P.J. (2010). What causes autism? Exploring the environmental contribution. Curr. Opin. Pediatr..

[14] Hallmayer J., Cleveland S., Torres A., Phillips J., Cohen B., Torigoe T., Miller J., Fedele A., Collins J., Smith K. (2011). Genetic heritability and shared environmental factors among twin pairs with autism. Arch. Gen. Psychiatry.

[15] James S.J., Zimmerman A.W. (2008). Oxidative stress and the metabolic pathology of autism. Autism: Current Theories and Evidence.

[16] Quig D. (1998). Cysteine metabolism and metal toxicity. Altern. Med. Rev..

[17] Mendola P., Selevan S.G., Gutter S., Rice D. (2002). Environmental factors associated with a spectrum of neurodevelopmental deficits. Ment. Retard. Dev. Disabil. Res. Rev..

[18] CDC’s Advisory Committee on Childhood Lead Poisoning Prevention (2007). Interpreting and managing blood lead levels <10 microg/dL in children and reducing childhood exposures to lead: recommendations of CDC’s Advisory Committee on Childhood Lead Poisoning Prevention. MMWR Recomm. Rep..

[19] Canfield R.L., Henderson C.R., Cory-Slechta D.A., Cox C., Jusko T.A., Lanphear B.P. (2003). Intellectual impairment in children with blood lead concentrations below 10 microg per deciliter. N. Engl. J. Med..

[20] American Academy of Pediatrics Committee on Environmental Health (2005). Lead exposure in children: Prevention, detection, and management. Pediatrics.

[21] Centers for Disease Control and Prevention (CDC) (2012). CDC Response to Advisory Committee on Childhood Lead Poisoning Prevention Recommendations in “Low Level Lead Exposure Harms Children: A Renewed Call of Primary Prevention”.

[22] Centers for Disease Control and Prevention (CDC) (2012). What Do Parents Need to Know to Protect Their Children?—Update on Blood Lead Levels in Children.

[23] Bellinger D.C. (2008). Very low lead exposures and children’s neurodevelopment. Curr. Opin. Pediatr..

[24] Ha M., Kwon H.J., Lim M.H., Jee Y.K., Hong Y.C., Leem J.H., Sakong J., Bae J.M., Hong S.J., Roh Y.M. (2009). Low blood levels of lead and mercury and symptoms of attention deficit hyperactivity in children: A report of the children’s health and environment research (CHEER). Neurotoxicology.

[25] Manser W.W., Khan M.A., Hasan Z. (1989). Trace element studies in Karachi populations. Part III: Blood copper, zinc, magnesium and lead levels in psychiatric patients with disturbed behaviour. J. Pak. Med. Assoc..

[26] Cohen D.J., Johnson W.T., Caparulo B.K. (1976). Pica and elevated blood lead level in autistic and atypical children. Am. J. Dis. Child.

[27] Fido A., Al-Saad S. (2005). Toxic trace elements in the hair of children with autism. Autism.

[28] Kern J.K., Grannemann B.D., Trivedi M.H., Adams J.B. (2007). Sulfhydryl-reactive metals in autism. J. Toxicol. Environ. Health A.

[29] Yorbik O., Kurt I., Hasimi A., Ozturk O. (2010). Chromium, cadmium, and lead levels in urine of children with autism and typically developing controls. Biol. Trace Elem. Res..

[30] Clark B., Vandermeer B., Simonetti A., Buka I. (2010). Is lead a concern in Canadian autistic children?. Paediatr. Child Health.

[31] Tian Y., Green P.G., Stamova B., Hertz-Picciotto I., Pessah I.N., Hansen R., Yang X., Gregg J.P., Ashwood P., Jickling G. (2011). Correlations of gene expression with blood lead levels in children with autism compared to typically developing controls. Neurotox. Res..

[32] Blaurock-Busch E., Amin O.R., Rabah T. (2011). Heavy metals and trace elements in hair and urine of a sample of arab children with autistic spectrum disorder. Maedica (Buchar).

[33] Blaurock-Busch E., Amin O.R., Dessoki H.H., Rabah T. (2012). Toxic metals and essential elements in hair and severity of symptoms among children with autism. Maedica (Buchar).

[34] Volk H.E., Lurmann F., Penfold B., Hertz-Picciotto I., McConnell R. (2013). Traffic-related air pollution, particulate matter, and autism. JAMA Psychiatry.

[35] Adams J.B., Audhya T., McDonough-Means S., Rubin R.A., Quig D., Geis E., Gehn E., Loresto M., Mitchell J., Atwood S. (2013). Toxicological status of children with autism *vs.* neurotypical children and the association with autism severity. Biol. Trace Elem. Res.

[36] Lakshmi Priya M., Geetha A. (2010). Level of trace elements (copper, zinc, magnesium and selenium) and toxic elements (lead and mercury) in the hair and nail of children with autism. Biol. Trace Elem. Res..

[37] El-Ansary A.K., Bacha A.B., Ayahdi L.Y. (2011). Relationship between chronic lead toxicity and plasma neurotransmitters in autistic patients from Saudi Arabia. Clin. Biochem..

[38] Alabdali A., Al-Ayadhi L., El-Ansary A. (2014). A key role for an impaired detoxification mechanism in the etiology and severity of autism spectrum disorders. Behav. Brain Funct..

[39] Knight C., Kaiser J., Lalor G.C., Robotham H., Witter J.V. (1997). Heavy metals in surface water and stream sediments in Jamaica. Environ. Geochem. Health.

[40] Lalor G.C. (1996). Geochemical mapping in Jamaica. Environ. Geochem. Health.

[41] Finster M.E., Gray K.A., Binns H.J. (2004). Lead levels of edibles grown in contaminated residential soils: A field survey. Sci. Total Environ..

[42] Wright V., Jones S., Omoruyi F.O. (2012). Effect of bauxite mineralized soil on residual metal levels in some post harvest food crops in Jamaica. Bull. Environ. Contam. Toxicol..

[43] Rahbar M.H., Samms-Vaughan M., Loveland K.A., Pearson D.A., Bressler J., Chen Z., Ardjomand-Hessabi M., Shakespeare-Pellington S., Grove M.L., Beecher C. (2012). Maternal and paternal age are jointly associated with childhood autism in Jamaica. J. Autism Dev. Disord..

[44] Rahbar M.H., Samms-Vaughan M., Loveland K.A., Ardjomand-Hessabi M., Chen Z., Bressler J., Shakespeare-Pellington S., Grove M.L., Bloom K., Pearson D.A. (2013). Seafood consumption and blood mercury concentrations in Jamaican children with and without autism spectrum disorders. Neurotox. Res..

[45] Rahbar M.H., Samms-Vaughan M., Ardjomand-Hessabi M., Loveland K.A., Dickerson A.S., Chen Z., Bressler J., Shakespeare-Pellington S., Grove M.L., Bloom K. (2012). The role of drinking water sources, consumption of vegetables and seafood in relation to blood arsenic concentrations of Jamaican children with and without Autism Spectrum Disorders. Sci. Total Environ..

[46] Rahbar M.H., Samms-Vaughan M., Dickerson A.S., Loveland K.A., Ardjomand-Hessabi M., Bressler J., Shakespeare-Pellington S., Grove M.L., Pearson D.A., Boerwinkle E. (2014). Blood manganese concentrations in Jamaican children with and without autism spectrum disorders. Environ. Health.

[47] Rahbar M.H., Samms-Vaughan M., Ma J., Bressler J., Loveland K.A., Ardjomand-Hessabi M., Dickerson A.S., Grove M.L., Shakespeare-Pellington S., Beecher C. (2014). Role of metabolic genes in blood arsenic concentrations of Jamaican children with and without autism spectrum disorder. Int. J. Environ. Res. Public Health.

[48] Rahbar M.H., Samms-Vaughan M., Dickerson A.S., Loveland K.A., Ardjomand-Hessabi M., Bressler J., Shakespeare-Pellington S., Grove M.L., Boerwinkle E. (2015). Factors associated with blood lead concentrations of children in Jamaica. J. Environ. Sci. Health A Tox. Hazard Subst. Environ. Eng..

[49] American Psychiatric Association (2000). Diagnostic and Statistical Manual of Mental Disorders, Fourth Edition Text Revision (DSM-IV-TR).

[50] Schopler E., Reichler R., DeVellis R., Daly K. (1980). Toward objective classification of childhood autism: Childhood Autism Rating Scale (CARS). J. Autism Dev. Disord..

[51] Lord C., Risi S., Lambrecht L., Cook E.H., Leventhal B.L., DiLavore P.C., Pickles A., Rutter M. (2000). The autism diagnostic observation schedule-generic: A standard measure of social and communication deficits associated with the spectrum of autism. J. Autism Dev. Disord..

[52] Lord C., Pickles A., McLennan J., Rutter M., Bregman J., Folstein S., Fombonne E., Leboyer M., Minshew N. (1997). Diagnosing autism: Analyses of data from the autism diagnostic interview. J. Autism Dev. Disord..

[53] Rutter M., Bailey A., Lord C. (2003). SCQ: The Social Communication Questionnaire. Manual.

[54] Mulligan A., Richardson T., Anney R., Gill M. (2009). The Social Communication Questionnaire in a sample of the general population of school-going children. Irish J. Med. Sci..

[55] Matte T.D., Figueroa J.P., Ostrowski S., Burr G., Jackson-Hunt L., Keenlyside R.A., Baker E.L. (1989). Lead poisoning among household members exposed to lead-acid battery repair shops in Kingston, Jamaica. Int. J. Epidemiol..

[56] Matte T.D., Figueroa J.P., Ostrowski S., Burr G., Jackson-Hunt L., Baker E.L. (1991). Lead exposure from conventional and cottage lead smelting in Jamaica. Arch. Environ. Contam. Toxicol..

[57] Gangoso L., Alvarez-Lloret P., Rodriguez-Navarro A.A., Mateo R., Hiraldo F., Donazar J.A. (2009). Long-term effects of lead poisoning on bone mineralization in vultures exposed to ammunition sources. Environ. Pollut..

[58] Agency for Toxic Substances and Disease Registry (ATSDR) (2007). Toxicological Profile for Lead.

[59] Sanna E., Liguori A., Palmas L., Soro M.R., Floris G. (2003). Blood and hair lead levels in boys and girls living in two Sardinian towns at different risks of lead pollution. Ecotoxicol. Environ. Saf..

[60] Schutz A., Olsson M., Jensen A., Gerhardsson L., Borjesson J., Mattsson S., Skerfving S. (2005). Lead in finger bone, whole blood, plasma and urine in lead-smelter workers: Extended exposure range. Int. Arch. Occup. Environ. Health.

[61] Fukui Y., Miki M., Ukai H., Okamoto S., Takada S., Higashikawa K., Ikeda M. (1999). Urinary lead as a possible surrogate of blood lead among workers occupationally exposed to lead. Int. Arch. Occup. Environ Health.

[62] Hirata M., Yoshida T., Miyajima K., Kosaka H., Tabuchi T. (1995). Correlation between lead in plasma and other indicators of lead exposure among lead-exposed workers. Int. Arch. Occup. Environ. Health.

[63] Gulson B.L., Cameron M.A., Smith A.J., Mizon K.J., Korsch M.J., Vimpani G., McMichael A.J., Pisaniello D., Jameson C.W., Mahaffey K.R. (1998). Blood lead-urine lead relationships in adults and children. Environ. Res..

[64] Shimbo S., Zhang Z.W., Moon C.S., Watanabe T., Nakatsuka H., Matsuda-Inoguchi N., Higashikawa K., Ikeda M. (2000). Correlation between urine and blood concentrations, and dietary intake of cadmium and lead among women in the general population of Japan. Int. Arch. Occup. Environ. Health.

[65] Moon C.S., Zhang Z.W., Shimbo S., Watanabe T., Moon D.H., Lee C.U., Lee B.K., Ahn K.D., Lee S.H., Ikeda M. (1998). Evaluation of urinary cadmium and lead as markers of background exposure of middle-aged women in Korea. Int. Arch. Occup. Environ. Health.

[66] Moreira M.F., Neves E.B. (2008). Use of urine lead level as an exposure indicator and its relationship to blood lead. Cad. Saude Publica.

[67] Lalor G., Vutchkov M., Bryan S. (2007). Blood lead levels of Jamaican children island-wide. Sci. Total Environ..

[68] Tong I.S., Lu Y. (2001). Identification of confounders in the assessment of the relationship between lead exposure and child development. Ann. Epidemiol..

[69] Janszky I., Ahlbom A., Svensson A.C. (2010). The Janus face of statistical adjustment: Confounders versus colliders. Eur. J. Epidemiol..

[70] Hernan M.A., Hernandez-Diaz S., Werler M.M., Mitchell A.A. (2002). Causal knowledge as a prerequisite for confounding evaluation: An application to birth defects epidemiology. Am. J. Epidemiol..

[71] Hernandez-Diaz S., Wilcox A.J., Schisterman E.F., Hernan M.A. (2008). From causal diagrams to birth weight-specific curves of infant mortality. Eur. J. Epidemiol..

[72] SAS Institute Inc. (2011). SAS® 9.3.

[73] Al-Farsi Y.M., Waly M.I., Al-Sharbati M.M., Al-Shafaee M.A., Al-Farsi O.A., Al-Khaduri M.M., Gupta I., Ouhtit A., Al-Adawi S., Al-Said M.F. (2013). Levels of heavy metals and essential minerals in hair samples of children with autism in Oman: A case-control study. Biol. Trace Elem. Res..

[74] Fombonne E., Simmons H., Ford T., Meltzer H., Goodman R. (2003). Prevalence of pervasive developmental disorders in the British nationwide survey of child mental health. Int. Rev. Psychiatry.

[75] Victora C.G., Wagstaff A., Schellenberg J.A., Gwatkin D., Claeson M., Habicht J.P. (2003). Applying an equity lens to child health and mortality: More of the same is not enough. Lancet.

[76] Bhasin T.K., Schendel D. (2007). Sociodemographic risk factors for autism in a US metropolitan area. J. Autism Dev. Disord..

[77] Durkin M.S., Maenner M.J., Meaney F.J., Levy S.E., DiGuiseppi C., Nicholas J.S., Kirby R.S., Pinto-Martin J.A., Schieve L.A. (2010). Socioeconomic inequality in the prevalence of autism spectrum disorder: evidence from a U.S. cross-sectional study. PLoS One.

[78] Hertz-Picciotto I., Green P.G., Delwiche L., Hansen R., Walker C., Pessah I.N. (2010). Blood mercury concentrations in CHARGE Study children with and without autism. Environ. Health Perspect..

[79] King M.D., Bearman P.S. (2011). Socioeconomic status and the increased prevalence of autism in California. Am. Sociol. Rev..

[80] Sasanfar R., Haddad S., Tolouei A., Ghadami M., Yu D., Santangelo S. (2010). Paternal age increases the risk for autism in an Iranian population sample. Mol. Autism.

[81] Croen L.A., Grether J.K., Hoogstrate J., Selvin S. (2002). The changing prevalence of autism in California. J. Autism Dev. Disord..

[82] Fountain C., King M.D., Bearman P.S. (2011). Age of diagnosis for autism: Individual and community factors across 10 birth cohorts. J. Epidemiol. Community Health.

[83] Windham G.C., Anderson M.C., Croen L.A., Smith K.S., Collins J., Grether J.K. (2011). Birth prevalence of autism spectrum disorders in the San Francisco Bay area by demographic and ascertainment source characteristics. J. Autism Dev. Disord..

[84] Liptak G.S., Benzoni L.B., Mruzek D.W., Nolan K.W., Thingvoll M.A., Wade C.M., Fryer G.E. (2008). Disparities in diagnosis and access to health services for children with autism: Data from the National Survey of Children’s Health. J. Dev. Behav. Pediatr..

[85] Larsson H.J., Eaton W.W., Madsen K.M., Vestergaard M., Olesen A.V., Agerbo E., Schendel D., Thorsen P., Mortensen P.B. (2005). Risk factors for autism: Perinatal factors, parental psychiatric history, and socioeconomic status. Am. J. Epidemiol..

[86] Naess O., Piro F.N., Nafstad P., Smith G.D., Leyland A.H. (2007). Air pollution, social deprivation, and mortality: A multilevel cohort study. Epidemiology.

[87] Ahamed M., Verma S., Kumar A., Siddiqui M.K. (2010). Blood lead levels in children of Lucknow, India. Environ. Toxicol..

[88] Iriani D.U., Matsukawa T., Tadjudin M.K., Itoh H., Yokoyama K. (2012). Cross-sectional study on the effects of socioeconomic factors on lead exposure in children by gender in Serpong, Indonesia. Int. J. Environ. Res. Public Health.

[89] Gale N.L., Adams C.D., Wixson B.G., Loftin K.A., Huang Y.W. (2002). Lead concentrations in fish and river sediments in the old lead belt of Missouri. Environ. Sci. Technol..

[90] Gale N.L., Adams C.D., Wixson B.G., Loftin K.A., Huang Y.W. (2004). Lead, zinc, copper, and cadmium in fish and sediments from the Big River and Flat River Creek of Missouri’s Old Lead Belt. Environ. Geochem. Health.

[91] Meador J.P., Ernest D.W., Kagley A.N. (2005). A comparison of the non-essential elements cadmium, mercury, and lead found in fish and sediment from Alaska and California. Sci. Total Environ..

[92] Rahbar M.H., White F., Agboatwalla M., Hozhabri S., Luby S. (2002). Factors associated with elevated blood lead concentrations in children in Karachi, Pakistan. Bull. World Health Organ.

[93] Valicenti-McDermott M., McVicar K., Rapin I., Wershil B.K., Cohen H., Shinnar S. (2006). Frequency of gastrointestinal symptoms in children with autistic spectrum disorders and association with family history of autoimmune disease. J. Dev. Behav. Pediatr..

[94] Horvath K., Perman J.A. (2002). Autistic disorder and gastrointestinal disease. Curr. Opin. Pediatr..

[95] Ibrahim S.H., Voigt R.G., Katusic S.K., Weaver A.L., Barbaresi W.J. (2009). Incidence of gastrointestinal symptoms in children with autism: A population-based study. Pediatrics.

[96] Rosenberg R.E., Daniels A.M., Law J.K., Law P.A., Kaufmann W.E. (2009). Trends in autism spectrum disorder diagnoses: 1994–2007. J. Autism Dev. Disord..

